# Caudal block vs. transversus abdominis plane block for pediatric surgery: a systematic review and meta-analysis

**DOI:** 10.3389/fped.2023.1173700

**Published:** 2023-05-30

**Authors:** Michael Hafeman, Seth Greenspan, Emiliya Rakhamimova, Zhaosheng Jin, Robert P. Moore, Ehab Al Bizri

**Affiliations:** ^1^ Department of Anesthesiology, Stony Brook University Renaissance School of Medicine, Stony Brook, NY, United States; ^2^ Stony Brook University Renaissance School of Medicine, Stony Brook, NY, United States; ^3^ Division of Pediatric Anesthesiology, Department of Anesthesiology, Stony Brook University Renaissance School of Medicine, Stony Brook, NY, United States

**Keywords:** acute postoperative pain, nerve block, anesthesia, caudal, pediatrics, abdominal muscles, inguinal hernia, genitourinary surgery

## Abstract

**Background:**

The caudal block and transversus abdominis plane block (TAP) are commonly used in combination with general anesthesia for pediatric lower abdominal, inguinal, and genitourinary surgeries. There is limited data directly comparing the impact of these techniques on recovery. In this meta-analysis, we compare the duration of postoperative analgesia between these two techniques.

**Objective:**

This review examined the duration of analgesia in pediatric patients (age 0–18 years) undergoing surgery who received caudal or TAP block after induction of general anesthesia. The primary outcome was duration of analgesia, defined as the time to first rescue analgesic dose. Secondary outcomes included number of rescue analgesic doses, acetaminophen usage within 24 h postoperatively, 24 h pain score area under the curve, and postoperative nausea and vomiting.

**Evidence review:**

We systematically searched Pubmed, Central, EMBASE, CINAHL, Google Scholar, Web of Science citation index, the US clinical trials register, and abstracts from prominent 2020–2022 anesthesia conferences for randomized controlled trials that compared these blocks and reported analgesia duration.

**Findings:**

Twelve RCTs inclusive of 825 patients were identified. TAP block was associated with longer analgesia duration (Mean difference = 1.76 h, 95% CI: 0.70–2.81, *p* = 0.001) and reduced doses of rescue analgesic within 24 h (Mean difference = 0.50 doses, 95% CI: 0.02–0.98, *p* = 0.04). No statistically significant differences were detected in other outcomes.

**Conclusion:**

This meta-analysis suggests that TAP block provides greater duration of analgesia than caudal block after pediatric surgeries. TAP block was also associated with fewer rescue analgesic doses in the first 24 h without increased pain scores.

**Systematic review registration:**

https://www.crd.york.ac.uk/prospero/display_record.php?RecordID=380876, identifier: CRD42022380876.

## Introduction

Lower abdominal, inguinal, and genitourinary surgeries are common in children and analgesia is a key aspect of perioperative care. Improvements in technology have led to the adoption of several regional anesthesia techniques in diverse clinical settings (1). There is limited comparative efficacy data about the impact of different regional techniques on recovery. This systematic review and meta-analysis sought to compare the caudal block (CB) to the transversus abdominis plane block (TAP) for these pediatric surgeries.

The caudal block is a widely used technique in which a needle traverses the sacral hiatus to deposit local anesthetic into the epidural space. It has traditionally been performed with a landmark-based technique, but ultrasound guidance is occasionally used (2, 3). While studies have shown it to be an effective technique for intraoperative analgesia in surgeries below the umbilicus, its utility may be limited by a short duration of analgesia (4). Additionally, although complications are rare and usually minor, they may be seven times more common in central vs. peripheral regional anesthesia in children and the potential for greater harm exists (5, 6). Caudal blocks may also result in motor blockade or urinary retention (7), which can potentially lengthen recovery. Additionally, caudal blocks can be difficult to place with an approximate 1% failure rate in data reported from a high-volume academic cohort (6).

The transversus abdominis plane block is an interfascial plane block technique in which local anesthetic is deposited between the internal oblique and transversus abdominis muscles, typically under ultrasound guidance (8). Several approaches have been described for this block, but they all involve deposition of anesthetic in this interfascial layer and may provide analgesia from the T10 to the L1 dermatomes (8, 9). The technique and related fascial plane blocks have become increasingly popular with widespread adoption of ultrasound technology for pediatric practice (1). Complications of the TAP block are rare but there is the potential for local anesthetic systemic toxicity and there are case reports of liver trauma and bowel hematoma when performed without ultrasound guidance (10, 11). Analgesia from the TAP block may last up to 48 h (12). This supposed long duration may be due to a longer lasting depot of local anesthetic at the injection site (13), compared to the great vascularity surrounding the epidural space which has been shown to decrease the duration of epidural analgesia (14).

While both techniques can be safely and successfully applied, there is limited data specifically related to the impact on the quality of postoperative analgesia, recovery, and efficiency of care when TAP block or caudal block are part of multimodal analgesia for lower abdominal surgeries in children. Such information is vitally important in an era increasingly focused on cost-effective patient-centered care that can resonate beyond the operating suite, such as enhanced recovery efforts (15, 16). This meta-analysis can help address this gap in the literature.

## Materials and methods

### Study objectives

The overall objective of this study was to compare analgesic efficacy of TAP block to caudal block in children undergoing lower abdominal, inguinal, or genitourinary surgery. The primary outcome was postoperative duration of analgesia achieved with either block, defined as the time from block or arrival in the recovery room until patients required a rescue analgesic dose. Secondary outcomes are the number of rescue doses, mean 24 h acetaminophen usage, 24 h area under the curve (AUC) pain score, and postoperative nausea and vomiting (PONV). This systematic review was registered under PROSPERO, ID CRD42022380876.

### Search strategy

This study was performed in accordance with the Preferred Reporting Items for Systematic Reviews and Meta-analysis (PRISMA) statement (17). Two authors independently searched for references, and any discrepancies were reconciled by the authors. We searched the terms “(Anesthesia, Caudal OR Caudal Anesthesia OR Sacral Epidural Anesthesia OR Caudal Block)” AND “(TAP Block OR Transversus Abdominis)” AND (“Pediatrics” OR Pediatrics OR Children) AND “(Surgery)” in PubMed, Central, EMBASE, CINAHL, Google Scholar, Web of Science citation index, and the US clinical trials register. Additionally, we manually searched the abstracts from prominent anesthesia conferences from the past three years. The search was completed on December 13, 2022.

### Study selection criteria

Two authors independently reviewed the title and abstract of the references compiled from the above search criteria and filtered through them based on the following criteria:

*Patient population:* The population consists of all pediatric patients (age ≤18) undergoing surgery who received either a caudal or TAP block immediately after induction of general anesthesia.

*Study design:* This meta-analysis compiled data exclusively from randomized controlled trials (RCTs).

*Intervention:* Pediatric patients who received a TAP block for postoperative analgesia. This meta-analysis excluded studies where patients received multiple blocks or studies that compared different medications administered for the blocks.

*Control:* Pediatric patients who received a caudal epidural block for postoperative analgesia. This meta-analysis excluded studies where patients received multiple blocks or studies that compared different medications administered for the blocks.

*Outcomes:* For inclusion in our study, an RCT must include the primary outcome of duration of analgesia.

### Data extraction

Data was extracted from each study onto a standardized form and was independently verified by a second author. The data extracted from each study included study title and author, number of participants in each experimental group, primary outcome (duration of analgesia), pain score at which rescue analgesia was administered, secondary outcomes (number of rescue doses, mean 24 h acetaminophen usage, 24 h area AUC pain score, and PONV), rescue analgesia medication and dosage, parent satisfaction, and other adverse events if reported, including urinary retention, hypotension, bradycardia, and block failure. We contacted the RCT authors when data was incomplete or reported with median and interquartile range. If the study authors did not reply, we derived the mean and standard deviation by assuming the normal distribution in accordance with Cochrane methods (18).

Risk of bias assessment was also performed and independently verified by two study authors in accordance with the RoB 2, a Cochrane risk-of-bias tool designed for randomized controlled trials (19). We examined the risk of bias in the RCTs in five domains: randomization and concealment of allocation, deviations form allocated interventions, completeness of outcome data, measurement bias, and the selection of reported results. Based on the summation of the study's risk of bias from these five domains, each study was assessed to be either “low risk,” “some concerns,” or “high risk” [of bias].

### Statistical analyses

We conducted meta-analysis for all outcomes. The data was analyzed using Review Manager V5.3. (Cochrane Collaboration, Copenhagen). For continuous variables, we used inverse-variance method to calculate the mean difference (MD) for outcomes that have clinically relevant effect sizes such as length of analgesia. For dichotomous variables, we calculated the risk ratios (RRs) by the Mantel-Haenszel method. Due to the inherent heterogeneous nature of block performance by different practitioners, random effect model was used in the analysis. We also conducted sensitivity analyses on the primary outcome (duration of analgesia) by excluding high-risk-of-bias studies. Small study effect was assessed using Egger's regression, and publication bias was assessed using Duval and Tweedie's trim and fill; both were done using the statistical package provided by Suurmond et al. (20). For all outcomes, the statistical significance was set to *p* < 0.05 and with 95% confidence intervals. We used GRADEpro Guideline Development Tool (GRADEpro GDT, McMaster University, 2015) to assess the quality of the meta-analysis findings, presented in Supplementary Table S1.

## Results

### Description of included studies

**Figure 1:**
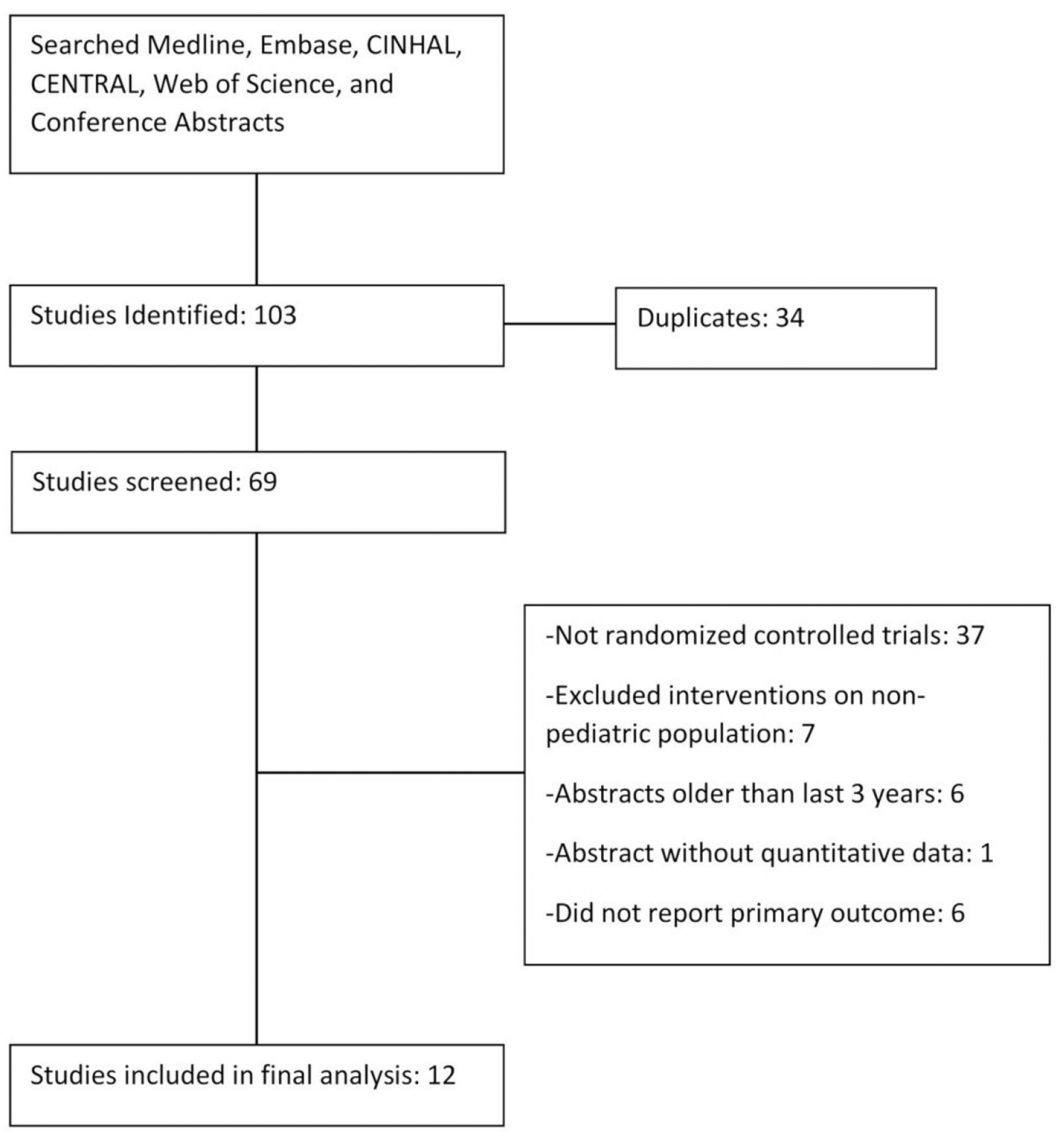
Search flow chart.

**Figure 2:**
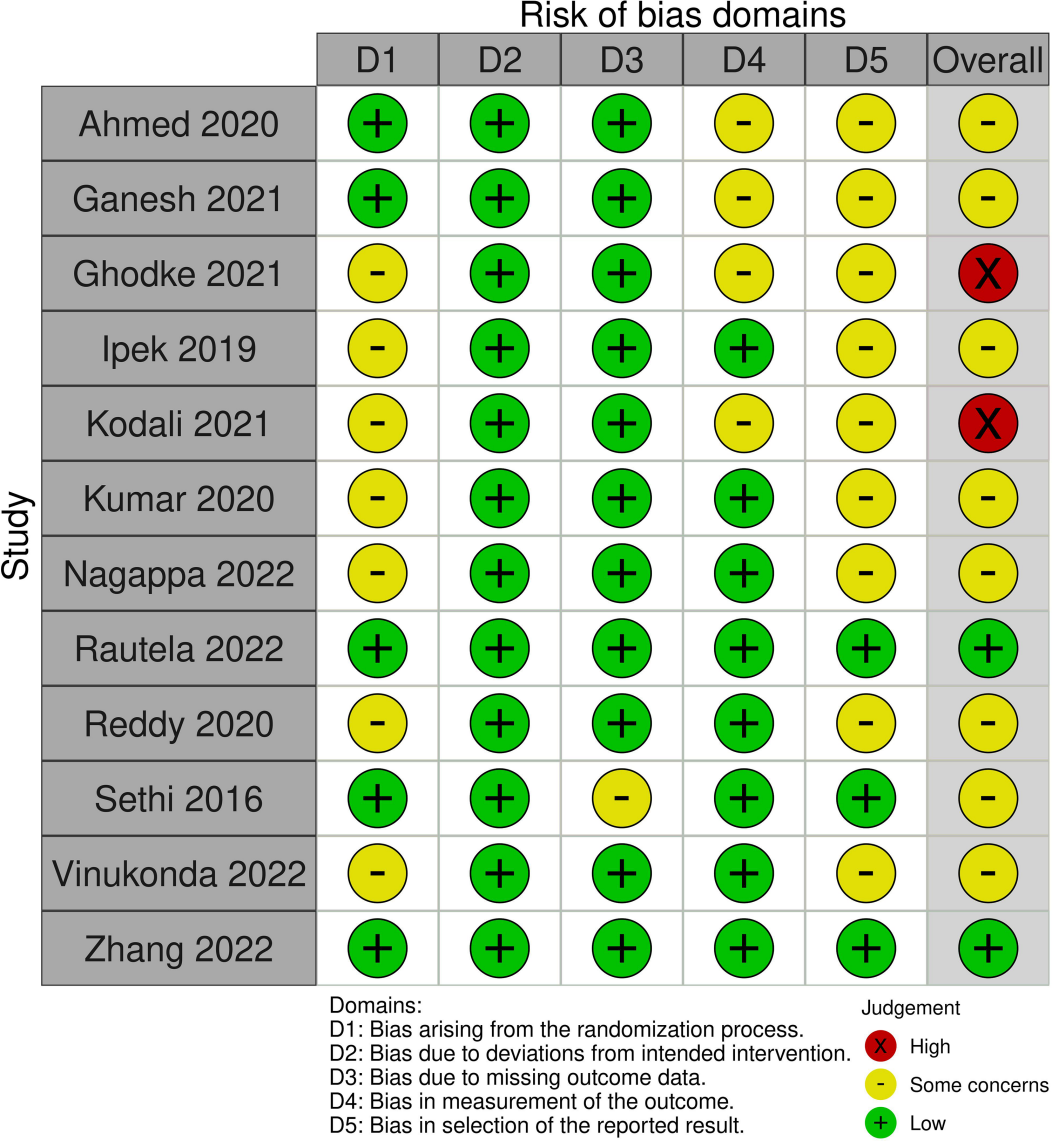
Risk of bias assessment for included randomized controlled trials.

The literature search identified a total of 69 unique studies. In the screening process, 57 of these studies were excluded and 12 were included in the meta-analysis (Figure 1) (21–32). Justification for exclusion of studies is provided in Supplementary Table S2. Characteristics of the included studies are listed in Table 1. A summary of findings is reported in Table 2. The risk of bias (RoB) assessment is reported in Figure 2, with the randomization process and selection of reported results being the most common sources of potential bias. Justification for RoB assessment is provided in Supplementary Table S3.

**Table 1:** Characteristics of included studies.

Study	Patient age (years)	Surgeries included	Block	Number of patients (n)	Medications administered in block	Pain score at which rescue analgesia was administered	Rescue analgesia dose
Ahmed 2020 (21)	2–6	Elective lower abdominal surgeries	Caudal	20	0.5% bupivacaine (dose not specified)	Not specified	IV paracetamol (dose not specified)
			TAP	20	0.5% bupivacaine (dose not specified)		
Ganesh 2021 (22)	2–7	Infraumbilical surgeries	Caudal	25	1 ml/kg 0.2% ropivacaine	FLACC > 3	IV acetaminophen 20 mg/kg, then IV fentanyl 1mcg/kg if pain persisted
			TAP	25	0.5 ml/kg 0.2% ropivacaine		
Ghodke 2021 (23)	1–8	Elective extraperitoneal lower abdominal wall surgeries	Caudal	25	1 ml/kg 0.2% levobupivacaine with 1 mg/kg dexamethasone	FLACC > 3	IV acetaminophen 15 mg/kg
			TAP	25	0.5 ml/kg 0.2% levobupivacaine with 1 mg/kg dexamethasone		
Ipek 2019 (24)	0.5–14	Elective unilateral lower abdominal wall surgery	Caudal	30	0.5 ml/kg 0.25% bupivacaine	POAS > 5	IV acetaminophen 10 mg/kg
			TAP	29	0.5 ml/kg 0.25% bupivacaine		
Kodali 2021 (25)	0.5–8	Inguinal hernia repair	Caudal	31	1 ml/kg 0.25% bupivacaine	FLACC > 4	IV acetaminophen 7.5 mg/kg
			TAP	31	0.5 ml/kg 0.25% bupivacaine		
Kumar 2020 (26)	2–8	Inguinal hernia repair	Caudal	56	1 ml/kg 0.2% ropivacaine	CHEOPS ≥ 6	PO acetaminophen 10 mg/kg
			TAP	56	0.5 ml/kg 0.2% ropivacaine		
Nagappa 2022 (27)	12–18	Laparoscopic appendectomy	Caudal	30	1 ml/kg 0.2% ropivacaine	VAS > 3	IV tramadol 1 mg/kg
			TAP	30	1 ml/kg 0.2% ropivacaine		
Rautela 2022 (28)	3–10	Elective unilateral infraumbilical surgery	Caudal	40	0.75 ml/kg 0.25% bupivacaine	MOPS ≥ 4	PO acetaminophen 15 mg/kg
			TAP	40	0.5 ml/kg 0.25% bupivacaine		
Reddy 2021 (29)	2–10	Lower abdominal surgery	Caudal	31	1 ml/kg bupivacaine with 1mcg/kg dexmedetomidine	FLACC > 4	IV acetaminophen 15 mg/kg
			TAP	31	0.5 ml/kg bupivacaine with 1mcg/kg dexmedetomidine		
Sethi 2016 (30)	2–6	Unilateral lower abdominal surgery	Caudal	36	0.75 ml/kg 0.25% bupivacaine	FLACC ≥ 3	IV fentanyl 1mcg/kg during first 2 h, PO acetaminophen 20 mg/kg thereafter
			TAP	34	0.5 ml/kg 0.25% bupivacaine		
Vinukonda 2022 (31)	2–8	Elective open unilateral inguinal hernia repair	Caudal	30	1 ml/kg 0.2% ropivacaine	CHEOPS > 6	IV acetaminophen 15 mg/kg
			TAP	30	0.5 ml/kg 0.2% ropivacaine		
Zhang 2022 (32)	1–12	Laparoscopic genitourinary or general surgery	Caudal	60	1 ml/kg 0.2% ropivacaine	FLACC > 4	IV tramadol 1 mg/kg
			TAP	60	1 ml/kg 0.2% ropivacaine		

FLACC, face, legs, activity, cry, consolability scale; POAS, pediatric objective pain scale; CHEOPS, children Hospital of Eastern Ontario pain scale; VAS, visual analog scale, MOPS, modified objective pain scale.

**Table 2:** Summary of findings.

Outcomes	TAP block mean or risk	Caudal block mean or risk	Effect size [95% confidence interval]	Number of participants (studies)	Quality or certainty of the evidence (GRADE)	Comments
Duration of analgesia (h)	7.55	5.79	**MD = 1.76 h [0.70–2.81]**	825 (12 studies)	⊕⊖⊖⊖ **Very low**	Significant heterogeneity, concerns of publication bias and small study effect
Number of rescue analgesic doses in first 24 h	1.64	2.14	**MD = −0.50 [−0.02 to −0.98]**	453 (6 studies)	⊕⊕⊖⊖ **Low**	Significant heterogeneity
Acetaminophen administered in first 24 h (mg/kg)	17.52	23.14	MD = −5.60 [−3.41 to 14.62]	503 (7 studies)	⊕⊕⊖⊖ **Low**	Significant heterogeneity
Pain score AUC in first 24 h	54.00	69.94	MD = −15.93 [−37.69 to 5.82]	526 (7 studies)	⊕⊖⊖⊖ **Very low**	Significant heterogeneity and concern of small study effect
Post operative nausea and vomiting	0.19	0.23	RR = 0.85 [0.47–1.55]	584 (8 studies)	⊕⊕⊕⊖ **Moderate**	Moderate heterogeneity

MD, mean difference; RR, risk ratio.

Population: Pediatric surgical patients.

Intervention: TAP block.

Comparator: Caudal block. Bolded values have statistical significance.

### Duration of analgesia

**Figure 3:**
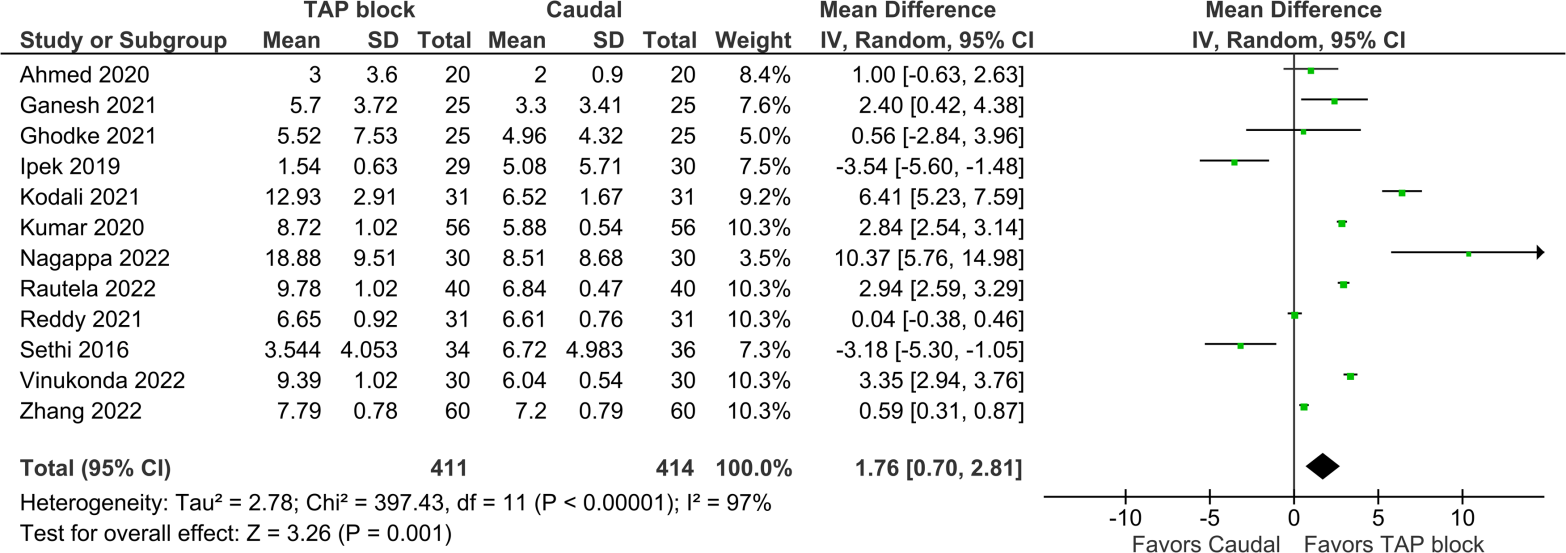
Forest plots of primary outcome of duration of analgesia (hours).

**Figure 4:**
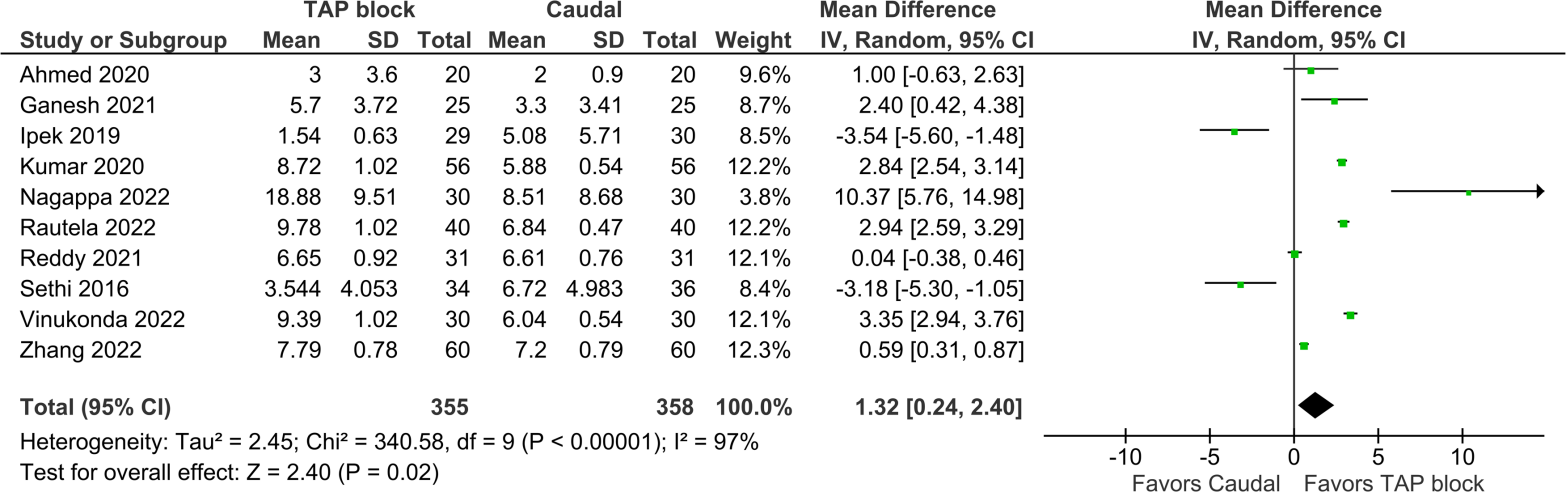
Forest plot of duration of analgesia, high risk of bias studies excluded.

Duration of analgesia was the primary outcome in this study and was reported in 12 trials. It was defined as the duration (from time of block or from time of arrival in recovery unit) before a dose of rescue analgesia was given; however, individual studies used different pain scores and scales to determine when this rescue should be administered (Table 1). Pooled results inclusive of 825 patients showed that the TAP block was favored [Mean difference (MD) = 1.76 h, 95% CI: 0.70–2.81, *I*^2^ = 97%, *p* = 0.001, Figure 3]. Excluding the high risk studies (per the RoB assessment) did not significantly alter the conclusion (MD = 1.32, 95% CI: 0.24–2.4, Figure 4). Egger's regression suggested significant risk of small study effect (*p* < 0.01); while the trim and fill predicted three missing studies. Quality of evidence is very low on account of the high heterogeneity and significant risk of publication bias.

### Number of rescue doses and 24 h acetaminophen usage

**Figure 5:**
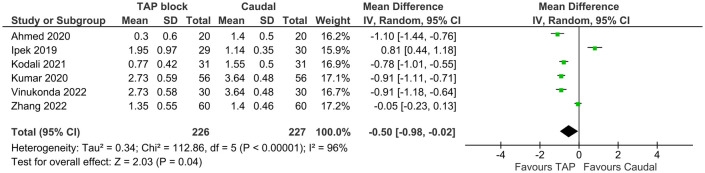
Forest plot of number of rescue analgesic doses in first 24 h.

Number of rescue doses was reported in six studies inclusive of 453 patients, with pooled results showing TAP block favored (MD = −0.50 doses, 95% CI: −0.02 to −0.98, *I*^2^ = 96%, *p* = 0.04, Figure 5). Egger's regression *p* = 0.42, trim and fill predicted no missing studies. The quality of evidence is low due to significant heterogeneity.

**Figure 6:**
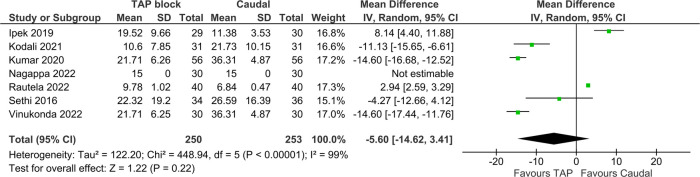
Forest plot of Acetaminophen usage in first 24 h (mg/kg).

Mean total acetaminophen in the first 24 h postoperatively was reported in seven studies inclusive of 503 patients, with pooled results showing no statistically significant difference [MD = −5.60 mg/kg (TAP block favored), 95% CI: −14.62−3.41, *I*^2^ = 99%, *p* = 0.22, Figure 6]. Egger's regression *p* = 0.41, trim and fill predicted no missing studies. The quality of evidence is low due to significant heterogeneity.

### Pain scores in first 24 h

**Figure 7:**
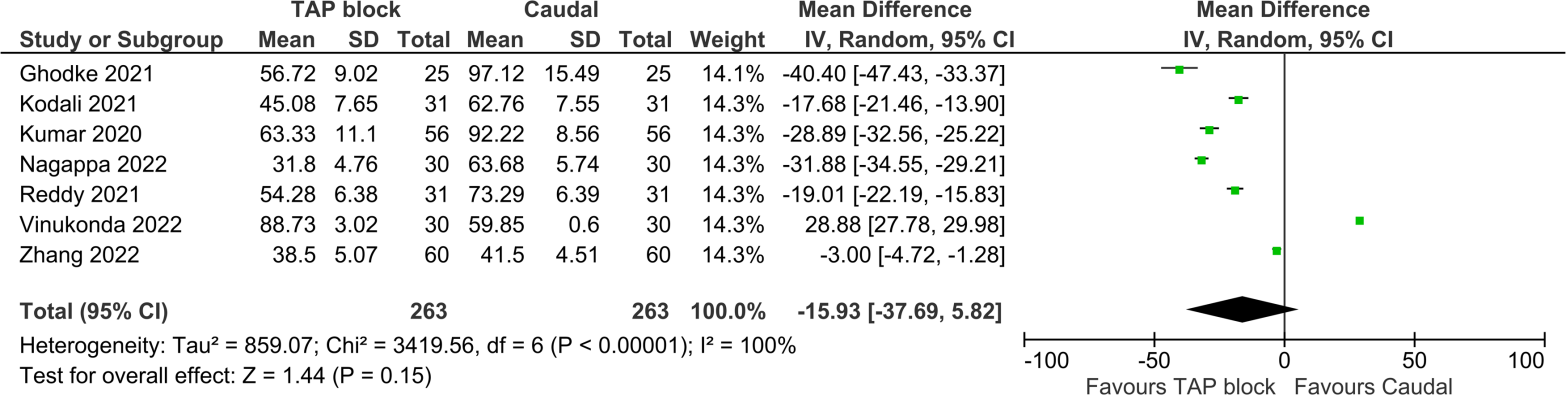
Forest plot of 24 h pain score AUC.

Of the 12 studies included in the primary analysis, seven studies inclusive of 526 patients reported pain scores up to 24 h postoperatively. Pooled results did not show a statistically significant difference between the pain score AUC of two blocks [MD = −15.93 (TAP block favored), 95% CI: −37.69–5.82, *I*^2^ = 100%, *p* = 0.15, Figure 7]. However, the study by Vinukonda et al. was an outlier and was the only study favoring CB in this metric. Egger's regression *p* = 0.01, trim and fill predicted no missing studies. The quality of evidence is very low due to significant heterogeneity and concern of small study effect.

### Post-operative nausea and vomiting

**Figure 8:**
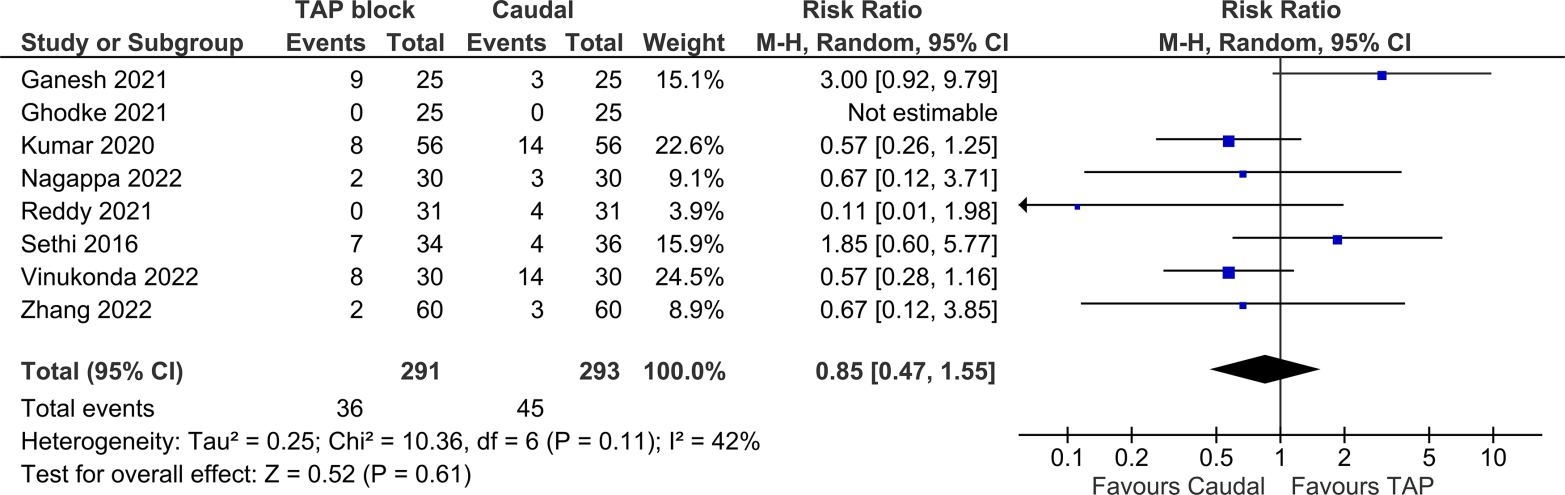
Forest plot of post operative nausea and vomiting.

Post-operative nausea and vomiting (PONV) was the only adverse outcome that was consistently reported, in a total of eight studies inclusive of 584 patients. Pooled analysis showed no statistically significant difference between the blocks [RR = 0.85 (TAP block favored), 95% CI: 0.47–1.55, *I*^2^ = 42%, *p* = 0.61, Figure 8]. Egger's regression *p* = 0.99, trim and fill predicted no missing studies. The quality of evidence moderate due to moderate heterogeneity. Of note, among the studies which reported PONV, Ganesh 2021 and Sethi 2016 were the only studies with a risk ratio favoring CB, and they were also the only studies to include IV fentanyl in the rescue analgesia regimen.

## Discussion

Pooled results demonstrated a significantly increased duration of analgesia of 1.76 h with techniques incorporating TAP block as compared to caudal blockade. This trend was associated with a statistically significant reduction in number of rescue analgesic doses in the first 24 h postoperatively (MD = −0.50 doses) with TAP block but no difference in the total weight-based acetaminophen in the first 24 h. This increased duration of analgesia was associated with a non-significant trend toward overall reduction in pain scores for the first 24 h (seen in six of seven analyzed studies). Additionally, there was a non-significant trend toward reduction in PONV amongst TAP block recipients. Parent/patient satisfaction was only reported in three of 12 studies and was not formally analyzed in this meta-analysis, however this metric also favored TAP block in all three studies. These results are thought-provoking and could have an immediate clinical impact while serving as a springboard for further research.

Analgesia is a key aspect of recovery from pediatric surgery and the addition of regional anesthesia has been particularly impactful. Regional techniques have been associated with reduced general anesthetic requirements with subsequent reduction in associated side effects and the potential for faster and smoother emergence, reduced opioid use, faster return of gut function, and reduced hormonal stress response (33). These benefits may be especially impactful for lower abdominal surgeries that are both commonly performed and associated with a non-trivial incidence of chronic pain after surgery (34). Both neuraxial and fascial plane blocks have been successfully applied in this context but there is limited data especially related to duration and quality of analgesia. This meta-analysis compares the impact of these techniques on the duration and impact of analgesia.

### Clinical implications

The application of TAP blocks to provide prolonged analgesia in comparison to caudal blockade without leading to an increase in postoperative pain scores or the need for rescue medications could support the expansion of regional techniques in pediatric practice at a time of significant change within the subspecialty (35, 36). In addition to serving as a reliable option for analgesia, the TAP block may be a more accessible technique relying on the transfer of skills commonly employed in adult practice to produce a block that is reliable and has a broad margin of safety. While no specific studies address comparative ease of pediatric vs. adult TAP block placement, pediatric anatomy may result in a technically simpler intervention due to favorable conditions to visualize anatomic landmarks and local anesthetic spread.

TAP blocks may have a greater margin of safety compared to caudal blockade especially when performed by non-subspecialty trained generalists. The application of fascial plane blocks obviates the risk of dural puncture, urinary retention, and neuraxial hematoma that can be associated with a neuraxial technique. They may have a greater window of safety related to placement-associated hematoma risk (37). Large studies have shown that complications are more frequent with neuraxial anesthesia and peripheral regional techniques may be preferred when appropriate (5, 38).

Similarly, TAP blocks may be placed more reliably, with lower rate of block failure or abandoned block. A large study by Polaner et al. showed only one block that was failed or abandoned out of 140 (0.7%). Of these 140 blocks, 92% were performed under ultrasound guidance. The caudal block has been more extensively studied. The same study by Polaner et al. examined over 6,000 caudal blocks, 97% with the landmark technique and 3% ultrasound guided, and found a complication rate of 3%. The majority of these complications (2%) were failed or abandoned blocks, but also reported were positive test doses, dural punctures, and vascular punctures (2). Another study of 750 caudal blocks in children without ultrasound guidance reported an overall success rate of 96%, with 70% successful on first attempt and 26% requiring multiple attempts (39). While the speed of placing a caudal block without ultrasound guidance may potentially be greater, this advantage would be negated by the significantly higher rate of failure, abandonment, and other complications.

This pooled analysis suggests a number of possible benefits of the significantly prolonged duration of analgesia associated with TAP block. Patients receiving TAP block received fewer rescue analgesia doses, and while not statistically significant, other possible benefits include a trend toward reduced pain scores and reduced PONV. Overall, this data combined with great ease of placement and a broad margin of safety support the application of TAP blocks for pediatric lower abdominal surgeries. It also suggests numerous areas for further research.

### Implications for further research

The pooled data support a significant increase in duration of analgesia when TAP block is applied as opposed to caudal blockade. The clinical implications of this difference and their impact on the perioperative care and overall quality of recovery are key questions to be addressed. Trends toward improved pain scores, reduced number of rescue doses, and PONV are suggestive of key areas for study. This information could be impactful for the design, implementation, and success of enhanced recovery after surgery (ERAS) pathways (15, 16, 40). The impact of prolonged duration of anesthesia on the progression to persistent pain is another key question for exploration. Although this study examines a wide variety of surgeries, further studies could elucidate the optimal surgeries for the TAP block, which provides mainly somatic analgesia and may be a poor choice for surgeries which involve extensive intraabdominal manipulation (41). Other fascial plane blocks, such as the erector spinae plane (ESP) block, may be further investigated as well. It has been suggested that the ESP block provides visceral analgesia (42, 43), and it has been successfully employed for a number of painful abdominal interventions (44, 45). Translational research could attempt to explain the mechanism for the different recovery profile between peripheral and neuraxial blocks. Specifically, are the observed benefits simply the product of different distribution and absorption of local anesthetic, or are they related to impact on the neurohormonal and inflammatory response to surgery?

### Strengths and limitations

Our pooled analysis is rooted in a thoughtful and systematic search process that included independent verification and extraction of data by two authors. This allowed for the analysis of 12 studies that enrolled a total of 825 patients. Results generated by this large cohort are important as it is difficult to complete large pediatric trials that are required to address questions related to recovery profiles. It can be challenging to enroll pediatric patients in clinical trials (46). Our process also allowed for the collection of data from several studies related to secondary outcomes including AUC for pain scores, rescue analgesia use, and incidence of PONV. This could provide insight into the differential impact of these techniques in the overall recovery profile. Pooling of data allowed for examination of these data points and is suggestive of the need for further research.

The primary limitation of this study is the heterogeneity of studies included. A number of different surgery types were included and performed on children of a variety of ages. Due to this heterogeneity, certainty of evidence ranged from low to very low. A more focused study on a particular age group or surgical population may reveal different results. There was also significant variability in the dosing of local anesthetic, adjuvants, and intraoperative opioid regimens (which were rarely reported). Additionally, included studies used different pain scales and thresholds for pain scores when defining their duration of analgesia and administering rescue medication. While this heterogeneity is a concern related to the quality of included studies, it is a clear reflection of the diversity of pediatric anesthesia practice and suggests that our primary outcome is the product of daily real-world conditions allowing for ease of clinical application. Similarly, there may be concern toward publication bias in the included studies. However, the large pooled number of included patients and overall support for the use of regional analgesia techniques in the context of lower abdominal surgeries (33) could blunt these concerns.

## Conclusion

Our large pooled analysis supports increased duration of analgesia with the application of TAP blocks as compared to caudal blockade for pediatric lower abdominal, inguinal, and genitourinary surgeries. Increased duration of analgesia was associated with fewer rescue analgesic doses and was not associated with increased pain scores. In fact, there were trends suggestive of reduced pain scores and reduced PONV when TAP was employed. Although this data has limitations and may not be applied universally, it generally supports usage of the TAP block, a technique with a wide margin of safety and ease of application.

## Data Availability

The original contributions presented in the study are included in the article/**Supplementary Material**, further inquiries can be directed to the corresponding author.
