# Supplementary material for: Caudal block vs. transversus abdominis plane block for pediatric surgery: a systematic review and meta-analysis
Source: Front Pediatr. 2023 May 30;11:1173700. doi: 10.3389/fped.2023.1173700 (PMC10265625; doi:10.3389/fped.2023.1173700)
Supplement: Supplementary file 1 [file Table1.docx]

Supplementary Material

Caudal Block Versus Transversus Abdominis Plane Block for Pediatric Surgery: A Systematic Review and Meta-Analysis

**Michael Hafeman, MD,* Seth Greenspan, BS, Emiliya Rakhamimova, BA, Zhaosheng Jin, MBBS, Robert P. Moore, MD, and Ehab Al Bizri, MD**

*** Correspondence:** Corresponding Author: [michaelrhafeman@gmail.com](mailto:michaelrhafeman@gmail.com)

Table S1: GRADE summary of the meta-analysis outcomes

| **Certainty assessment** | | | | | | | **№ of patients** | | **Effect** | | **Certainty** |
| --- | --- | --- | --- | --- | --- | --- | --- | --- | --- | --- | --- |
| **№ of studies** | **Study design** | **Risk of bias** | **Inconsistency** | **Indirectness** | **Imprecision** | **Other considerations** | **Caudal block** | **TAP block** | **Relative (95% CI)** | **Absolute (95% CI)** |  |
| Duration of analgesia | | | | | | | | | | | |
| 12 | randomized trials | not serious | very serious^a^ | not serious | not serious | publication bias strongly suspected ^b^ | 414 | 411 | - | **MD 1.76 Hours**  [TAP favored] (0.7 to 2.81) | ⨁◯◯◯ Very low |
| Number of rescue doses | | | | | | | | | | | |
| 6 | randomized trials | not serious | very serious^c^ | not serious | not serious | none | 227 | 226 | - | **MD -0.50 doses**  [TAP favored]  (-0.02 to -0.98) | ⨁⨁◯◯ Low |
| Weight adjusted acetaminophen dosage | | | | | | | | | | | |
| 7 | randomized trials | not serious | very serious^c^ | not serious | not serious | none | 253 | 250 | - | MD 5.6 mg/kg  [TAP favored]  (-3.41 to 14.62) | ⨁⨁◯◯ Low |
| Pain score area under the curve | | | | | | | | | | | |
| 7 | randomized trials | not serious | very serious^c^ | not serious | not serious | Small study effect strongly suspected^d^ | 263 | 263 | - | MD -15.93 [TAP favored]  (-37.69 to 5.82) | ⨁◯◯◯ Very low |
| Postoperative nausea and vomiting | | | | | | | | | | | |
| 8 | randomized trials | not serious | Serious^e^ | not serious | not serious | none | 45/293 (15.4%) | 36/291 (12.4%) | RR 0.85 (0.47 to 1.55) | 19 fewer per 1,000 (-66 to 68) | ⨁⨁⨁◯ Moderate |

**CI:** confidence interval; **MD:** mean difference; **RR:** risk ratio; **TAP**: transverse abdominis plane (block)

Explanations

a. Significant unexplained heterogeneity despite subgroup analysis.

b. Trim and Fill predicted 3 missing studies.

c. Significant unexplained heterogeneity.

d. Egger's regression concerning for small study effect

e. Moderate heterogeneity

Table S2: Description of excluded studies

| Study Name and Year | DOI | Title | Reason for exclusion |
| --- | --- | --- | --- |
| Alkayssi et al. 2022 | 10.14704/nq.2022.20.4.NQ22119 | Comparison between Caudal and TAP Blocks Post-Inguinal Surgery Analgesia in Children | Less than 24-hour follow-up period |
| Alsadek et al. 2015 | 10.1016/j.egja.2015.03.001 | Ultrasound guided TAP block versus ultrasound guided caudal block for pain relief in children undergoing lower abdominal surgeries | Less than 24-hour follow-up period |
| Bryskin et al. 2015 | 10.1213/ANE.0000000000000779 | Transversus Abdominis Plane Block Versus Caudal Epidural for Lower Abdominal Surgery in Children: A Double-Blinded Randomized Controlled Trial | Missing primary outcome |
| Fahmy et al. 2020 | 10.1093/qjmed/hcaa039.012 | Ultrasound Guided Transversus Abdominis Plane (TAP) Block versus Caudal Block in Pediatrics Undergoing Inguinal Hernia Repair | Abstract without quantitative data |
| Polat et al. 2022 | 10.1016/j.jpurol.2022.11.005 | Effects of ultrasound guided caudal epidural and transversus abdominis plane block on postoperative analgesia in pediatric inguinal hernia repair surgeries | Missing primary outcome |
| Sahin et al. 2017 | 10.15537/smj.2017.9.20505 | Comparison of 3 different regional block techniques in pediatric patients A prospective randomized single-blinded study | Erroneous data reported for primary outcome; authors did not clarify upon request. |
| Talukdar et al. 2020 | 10.4103/0019-5049.277904 | Comparison of caudal block and usg guided transversus abdominis plane block for analgesic efficacy in paediatric patients in lower abdominal surgeries-a randomised double blinded study | Missing primary outcome |

Table S3: Justification for Risk of Bias Assessment for Included RCTs

|  | Ahmed 2020 |
| --- | --- |
| Randomization process | Low risk |
| Justification | Computer generated randomization, sealed envelope |
| Deviation from intended intervention | Low risk |
| Justification | Blinding not described, no likely protocol deviations |
| Missing Outcome data | Low risk |
| Justification | Outcome data for all participants |
| Measurement of outcome | Some Concerns |
| Justification | Observer blinding not stated |
| Selection of reported results | Some concerns |
| Justification | Trial not registered |

|  | Ganesh 2021 |
| --- | --- |
| Randomization process | Low |
| Justification | Computer generated randomization, sealed envelope |
| Deviation from intended intervention | Low |
| Justification | Blinding not described, no likely protocol deviations |
| Missing Outcome data | Low risk |
| Justification | Outcome data for all participants |
| Measurement of outcome | Some Concerns |
| Justification | Observer blinding not stated |
| Selection of reported results | Some concerns |
| Justification | Trial not registered |

|  | Ghodke 2021 |
| --- | --- |
| Randomization process | Some concerns |
| Justification | No description of randomization or concealment |
| Deviation from intended intervention | Low risk |
| Justification | Observer blinding not stated, no likely protocol deviations |
| Missing Outcome data | Low risk |
| Justification | Outcome data for all participants |
| Measurement of outcome | Some concerns |
| Justification | Observer blinding not stated |
| Selection of reported results | Some concerns |
| Justification | Trial not registered |

|  | Ipek 2019 |
| --- | --- |
| Randomization process | Some concerns |
| Justification | No description of concealment |
| Deviation from intended intervention | Low risk |
| Justification | Adequate blinding |
| Missing Outcome data | Low risk |
| Justification | Outcome data for all participants |
| Measurement of outcome | Low Risk |
| Justification | Adequate blinding |
| Selection of reported results | Some concerns |
| Justification | Trial not registered |

|  | Kodali 2021 |
| --- | --- |
| Randomization process | Some concerns |
| Justification | No description of concealment |
| Deviation from intended intervention | Low risk |
| Justification | Observer blinding not stated, no likely protocol deviations |
| Missing Outcome data | Low risk |
| Justification | Outcome data for all participants |
| Measurement of outcome | Some concerns |
| Justification | Observer blinding not stated |
| Selection of reported results | Some concerns |
| Justification | Trial not registered |

|  | Kumar 2020 |
| --- | --- |
| Randomization process | Some concerns |
| Justification | No description of concealment |
| Deviation from intended intervention | Low risk |
| Justification | Adequate blinding |
| Missing Outcome data | Low risk |
| Justification | Outcome data for all participants |
| Measurement of outcome | Low risk |
| Justification | Adequate blinding |
| Selection of reported results | Some concerns |
| Justification | Trial not registered |

|  | Nagappa 2022 |
| --- | --- |
| Randomization process | Some concerns |
| Justification | No description of concealment |
| Deviation from intended intervention | Low risk |
| Justification | Adequate blinding |
| Missing Outcome data | Low risk |
| Justification | 3.33% of patients did not complete study |
| Measurement of outcome | Low risk |
| Justification | Adequate blinding |
| Selection of reported results | Some concerns |
| Justification | Trial not registered |

|  | Rautella 2022 |
| --- | --- |
| Randomization process | Low risk |
| Justification | Computer generated randomization, sealed envelope |
| Deviation from intended intervention | Low risk |
| Justification | Adequate blinding |
| Missing Outcome data | Low risk |
| Justification | Outcome data for all participants |
| Measurement of outcome | Low risk |
| Justification | Adequate blinding |
| Selection of reported results | Low risk |
| Justification | Trial registered on CTRI/2014/09/005023 |

|  | Reddy 2021 |
| --- | --- |
| Randomization process | Some concerns |
| Justification | No description of concealment |
| Deviation from intended intervention | Low Risk |
| Justification | Adequate blinding |
| Missing Outcome data | Low risk |
| Justification | Outcome data for all participants |
| Measurement of outcome | Low risk |
| Justification | Adequate blinding |
| Selection of reported results | Some concerns |
| Justification | Trial not registered |

|  | Sethi 2016 |
| --- | --- |
| Randomization process | Low risk |
| Justification | Computer generated randomization, sealed envelope |
| Deviation from intended intervention | Low risk |
| Justification | Adequate blinding |
| Missing Outcome data | Some concerns |
| Justification | 12.5% of patients did not complete study |
| Measurement of outcome | Low risk |
| Justification | Adequate blinding |
| Selection of reported results | Low risk |
| Justification | Trial registered on CTRI/2012/01/002387 |

|  | Vinukonda 2022 |
| --- | --- |
| Randomization process | Some concerns |
| Justification | No concealment |
| Deviation from intended intervention | Low risk |
| Justification | Adequate blinding |
| Missing Outcome data | Low risk |
| Justification | Outcome data for all participants |
| Measurement of outcome | Low risk |
| Justification | Adequate blinding |
| Selection of reported results | Some concerns |
| Justification | Trial not registered |

|  | Zhang 2022 |
| --- | --- |
| Randomization process | Low risk |
| Justification | Computer generated randomization, sealed envelope |
| Deviation from intended intervention | Low risk |
| Justification | Adequate blinding |
| Missing Outcome data | Low risk |
| Justification | Outcome data for all participants |
| Measurement of outcome | Low risk |
| Justification | Adequate blinding |
| Selection of reported results | Low risk |
| Justification | Trial registered at ChiCTR2000032580 |
